# Transcription Analysis of Recombinant *Trichoderma reesei* HJ-48 to Compare the Molecular Basis for Fermentation of Glucose and Xylose

**DOI:** 10.4014/jmb.2004.04007

**Published:** 2020-07-17

**Authors:** Jun Huang, Mei Lin, Shijie Liang, Qiurong Qin, Siming Liao, Bo Lu, Qingyan Wang

**Affiliations:** National Engineering Research Center for Non-Food Biorefinery, State Key Laboratory of Non-Food Biomass and Enzyme Technology, Guangxi Key Laboratory of Biorefinery, Guangxi Biomass Engineering Technology Research Center, Guangxi Academy of Sciences, Nanning 530007, P.R. China

**Keywords:** Transcription, *Trichoderma reesei*, fermentation

## Abstract

Profiling the transcriptome changes involved in xylose metabolism by the fungus *Trichoderma reesei* allows for the identification of potential targets for ethanol production processing. In the present study, the transcriptome of *T. reesei* HJ-48 grown on xylose versus glucose was analyzed using nextgeneration sequencing technology. During xylose fermentation, numerous genes related to central metabolic pathways, including xylose reductase (XR) and xylitol dehydrogenase (XDH), were expressed at higher levels in *T. reesei* HJ-48. Notably, growth on xylose did not fully repress the genes encoding enzymes of the tricarboxylic acid and respiratory pathways. In addition, increased expression of several sugar transporters was observed during xylose fermentation. This study provides a valuable dataset for further investigation of xylose fermentation and provides a deeper insight into the various genes involved in this process.

## Introduction

Lignocellulosic biomass, such as corn stover, sugarcane bagasse, and straw, are among the most attractive feedstocks for bioethanol production. Sugar derived from plant biomass is a mixture of hexoses and pentoses. The yeast species *Saccharomyces cerevisiae* is the most successful industrial microorganism for ethanol production due to its high ethanol productivity, high growth rate, as well as its high tolerance for ethanol and low pH [1]. Nevertheless, wild-type strains of *S. cerevisiae* are unable to utilize pentose sugars. To overcome this obstacle, a *S. cerevisiae* strain encoding a pathway from pentose-assimilating microorganisms has been engineered [2, 3]. Various steps, including the inefficient pentose transport into the cell and the imbalance of cellular redox, have been implicated in the limited metabolism of pentose sugars in engineered *S. cerevisiae* [4]. Nature has evolved numerous pentose-utilizing microorganisms in which pentose transport systems are present for pentose uptake, especially filamentous fungi [5].

Filamentous fungi are the primary source of cellulase and possess a remarkable capacity for extracellular protein production. With regard to commercial production of cellulases, *Trichoderma reesei* is known to be an abundant producer of cellulases and secretes a number of complex cellulases, such as CBH1 [6]. Because of its wide array of encoded cellulase proteins, *T. reesei* is able to efficiently digest lignocellulose. Previous studies have shown that *T. reesei* can convert lignocellulosic sugars, including pentose sugars, to ethanol [7]. Moreover, *T. reesei* possesses pentose-utilizing pathways to enable the use of pentose sugars for ethanol production. However, *T. reesei* also presents several limitations, such as low ethanol tolerance and low ethanol productivity, that hinder its application for commercial use [7]. To date, several *T. reesei* strains have been metabolically engineered for improved ethanol production [8, 9]. In a previous study, we showed that the ethanol production of the recombinant strain HJ48 was higher than the parent strain CICC40360, although the mechanisms responsible for the different characteristics of sugar utilization between these two strains remain unknown [9].

Genome-wide studies involving expressed sequence tags, cDNA microarrays, and transcriptomics have been carried out to study the various genes related to glucose metabolism [10, 11]. We previously performed a comprehensive metabolome analysis using transcriptomics between the recombinant strain HJ48 and the parent strain CICC40360 during fermentation with glucose, and demonstrated that a series of glycolysis enzymes are upregulated in the recombinant strain HJ48 under fermentation conditions [12]. Although these studies provide a metabolic analysis for the low ethanol productivity of CICC40360 compared to HJ48, little is known about transcriptional differences between aerobic glucose and xylose fermentation by the recombinant strain HJ48. In this research we investigated the genes involved in the high ethanol productivity when grown using either xylose or glucose as a sole carbon source medium in the HJ48 strain. This study provides a genome-wide analysis of the transcriptional landscape of *T. reesei* grown on xylose and glucose, and offers a basis for further studies on ethanol production using pentose sugars.

## Materials and Methods

### Strains and Fermentation

The recombinant *T. reesei* strain HJ48 was used in this study [12]. Fermentation of *T. reesei* was carried out as previously described [12].

### RNA Sequencing

Total RNA from samples was isolated using TRIzol (Invitrogen Life Technologies, USA) according to the manufacturer’s instructions, and treated with RNase-free DNase I (Tiangen, China) to remove any DNA contamination. Qubit RNA Assay Kit (Life Technologies, USA) and Nano 6000 Assay Kit (Agilent Technologies, USA) were used to check the RNA concentration and integrity. Three micrograms of RNA per sample was used for the RNA sample preparations. The sequencing libraries were created by NEBNext Ultra RNA Library Prep Kit for Illumina (NEB, USA). mRNA was purified from total RNA using Poly-T oligo-attached magnetic beads. RNA fragmentation and cDNA synthesis were performed according to a protocol from a previous study [13]. Library preparations were sequenced on an Illumina HiSeq 2500 platform, and 100 bp paired-end reads were generated. Clean data obtained in the FASTQ format were performed through in-house Perl scripts. Clean data were then analyzed by removing adapter, low-quality reads, and reads containing ploy-N from the raw data. The Q20, Q30, and GC content of the clean data were calculated. The *T. reesei* genome was downloaded from the interactive JGI Genome Portal [14]. The index of the reference genome was built using Bowtie v2.0.6. The paired-end clean reads were aligned to the reference genome using TopHat v2.0.9 [15]. Counts of the read numbers were mapped to each gene using HTSeq v0.6.1 [16]. The Reads Per Kilobase per Million mapped reads (RPKM) of each gene was calculated based on the length of the gene and read counts mapped to the gene [17].

### Data Analysis

EdgeR was used for each sequenced library via one scaling normalized factor to adjust the read counts. DEGSeq R package (1.12.0) was used to analysis differentially expressed genes of two conditions, a q-value (corrected *p*-value) < 5 and log2 (fold-change) >1 [18]. The *p*-value was adjusted according to the findings of a previous study [19]. Gene ontology (GO) enrichment analysis of differentially expressed genes was analyzed using the GOseq R package, in which the gene length bias was corrected. GO terms with the corrected *p*-value less than 0.05 were considered significantly enriched by differentially expressed genes [20]. The KOBAS software was used for statistical enrichment of differential gene expression in KEGG pathways [21].

### Real-Time Quantitative PCR (qPCR)

qPCR was used to check the expression of thirteen genes involved in different metabolism pathways. Reverse transcription was done using the PrimeScript RT Reagent Kit with gDNA Eraser (TaKaRa Japan) and qPCR was done using the TB Green Premix Ex Taq II Kit (TaKaRa) in Agilent Technologies AriaMx Real-Time PCR, each with three repeats. The relative expression levels of each gene were implemented using the 2^-ΔΔCT^ method, with the expression of glucose fermentation as the control and the expression of the tubulin gene as the internal standard. Primers for qPCR are listed in Table 1.

## Results

### Ethanol Fermentation

**Fig. 1:**
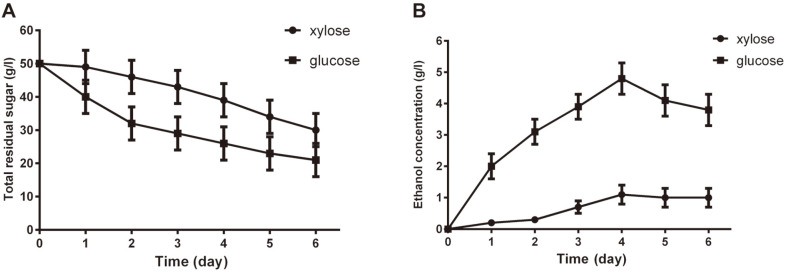
Time course of glucose consumption, xylose consumption (A), and ethanol production (B) by HJ48 under anaerobic conditions in GM medium containing 50 g/l glucose and XM medium containing 50 g/l xylose. The values are means of triplicate experiments. Error bars indicate standard deviations.

To study the effects of different carbon sources, specifically glucose and xylose, on ethanol fermentation, fermentation by the recombinant *T. reesei* strain HJ48 was performed anaerobically in fermentation medium (FM) supplied with 50 g/l glucose (GM) and 50 g/l xylose (XM). As shown in Figs. 1A and B, the recombinant strain HJ48 showed different fermentation modes depending on the carbon source of the fermentation medium. When the recombinant strain HJ48 was grown in GM on anaerobic conditions, the glucose was almost consumed by HJ48 after 72 h of cultivation. Meanwhile, an ethanol concentration of 4.8 ± 2 g/l was detected.

When xylose was provided as a sole carbon source, half of the xylose was consumed by HJ48 after 72 h of cultivation, demonstrating that the recombinant strain HJ48 consumes xylose at a slower rate than it does glucose after 72 h of cultivation. Additionally, an ethanol concentration of 1.1 g/l was detected. The maximum ethanol production of the recombinant strain HJ48 achieved in fermentation with XM was lower than that observed with GM.

### Transcriptome Analysis of *T. reesei* Strain HJ48 Growth on Xylose Versus Glucose

**Fig. 2:**
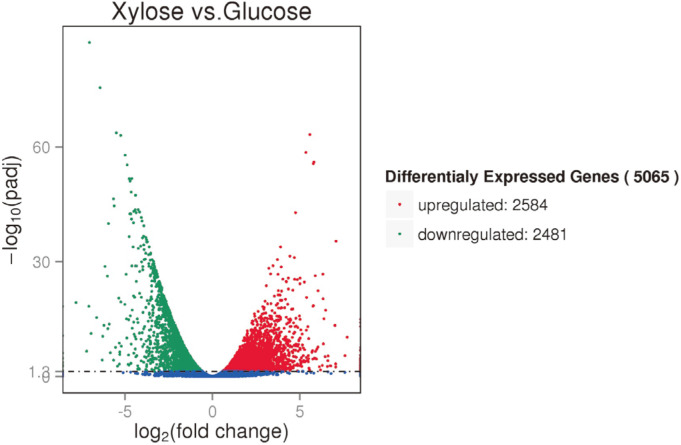
Overview of the differentially expressed genes between xylose and glucose.

The transcriptome of *T. reesei* growing on glucose and xylose was analyzed using RNA-sequencing after 72 h of fermentation under anaerobic conditions. The total RNA of the recombinant strain HJ48 was extracted, and RNA-sequencing was conducted using Illumina HiSeq2000. After removing low quality reads, clean reads (20.56 G) were obtained, and were used as the basis of all downstream studies. The sequence data produced in this study can be accessed [GEO: GSE84393]. Detailed information on the assembly process is shown in Table 2. Clean reads were mapped to the *T. reesei* genome. The normalized expression values of each annotated gene were calculated using FPKM (expected number of Fragments Per Kilobase of transcript sequence per Million base pairs sequenced). In this study, only the genes with FPKM >1 were recognized as potentially expressed. To study significantly upregulated and downregulated genes during growth on either glucose or xylose, only the genes with q-values less than 0.05 were selected for further analysis. Overall, 5,065 genes showed significantly different expression when grown on xylose compared to that on glucose. We observed that 2,584 genes were upregulated, whereas 2,481 genes were downregulated (Fig. 2). These results suggest that there were significant differences in *T. reesei* gene expression under glucose and xylose growth conditions.

**Fig. 3:**
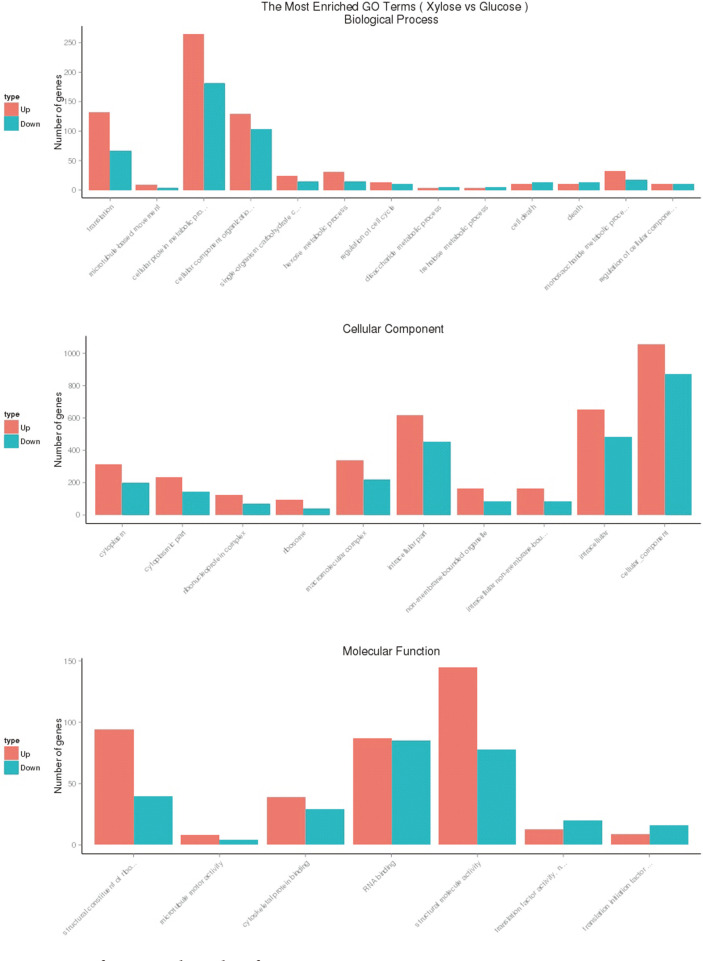
Histogram of gene ontology classification. Gene ontology (GO) enrichment analysis of differentially expressed genes was implemented by the goseq R package. The results are summarized in three main categories: biological process, cellular component, and molecular function.

**Fig. 4:**
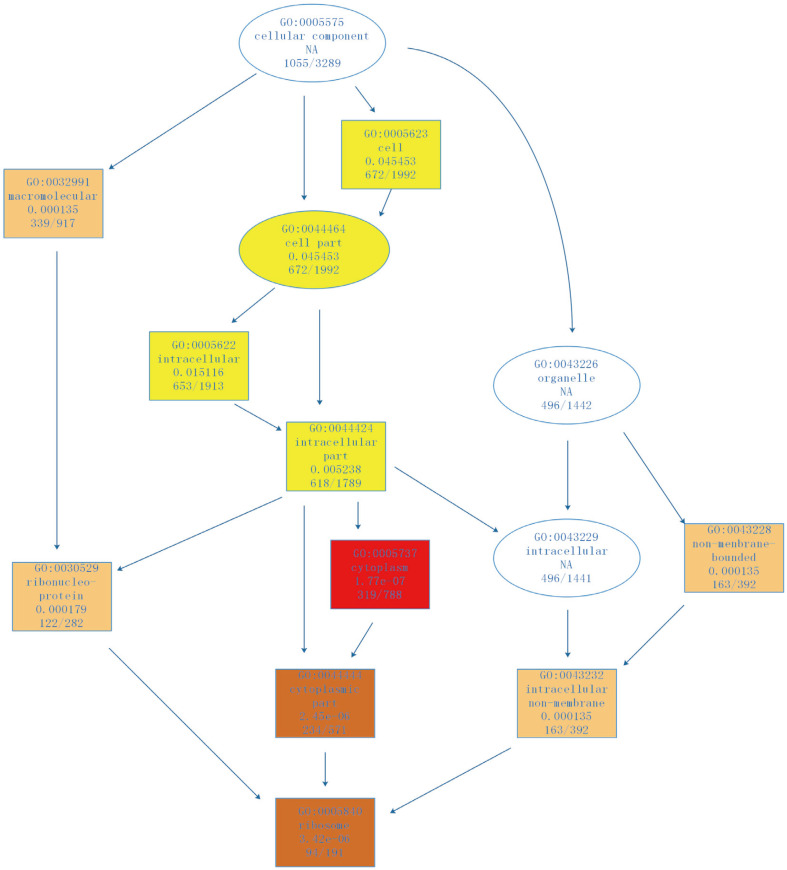
The relationship of upregulated GO terms involved in cellular components. Gene enrichment is represented by color, and the darker colors signify increased enrichment.

We next performed gene ontology (GO) term analysis to classify the functions of the differentially expressed genes. Surprisingly, we did not identify any significant enrichment in GO terms (*p* < 0.05) for the genes found to be downregulated during growth on glucose or xylose. Table 3 shows the GO terms (*p* < 0.05) found to be enriched for the genes that were significantly upregulated. Based on common biological properties, the identified genes could be classified into three groups: biological processes, cellular components, and molecular functions (Fig. 3). As shown in Fig. 3, the biological processes category included 13 subcategories, with 265 genes in the cellular protein metabolic process [GO:0044267], 130 genes in cellular component organization [GO:0016043], and 132 genes in translation [GO:0006412]. The cellular component category was split into 10 subcategories, in which 1,055 genes were shown to be involved in cellular components [GO:0005575], 653 in intracellular [GO:0005622], 618 in the intracellular part [GO:0044424], 337 in macromolecular complex [GO:0032991], 316 in cytoplasm [GO:0005737], and 243 genes in the cytoplasm part [GO:0044444]. The GO terms cytoplasm, structural constituent of ribosome, ribosome, ribonucleoprotein complex, and cellular protein metabolic process, were all found to be enriched within the group of upregulated genes involved in the cellular component (Fig. 4). The group of upregulated genes for cellular component seems to directly correlate to the efficiency of cell growth. The genes within the molecular function group were split into 7 subcategories, with 145 genes involved in structural molecule activity [GO:0005198], 94 genes in structural constituent of ribosome [GO:0003735], and 87 genes in RNA binding [GO:0003723].

In order to categorize the genes found to be differentially expressed into functional pathways between xylose and glucose utilized by the recombinant strain HJ48, the genes were classified based on KEGG enrichment using FDR ≤ 0.05. The upregulated expression genes between xylose and glucose utilized by the recombinant strain HJ48 were predominantly functionally categorized into 20 pathways, including ribosome [tre03010], proteasome [tre03050], peroxisome [tre04146], alanine, aspartate and glutamate metabolism [tre00250], metabolic pathways [tre01100], aminoacyl-tRNA biosynthesis [tre00970], valine, leucine and isoleucine degradation [tre00280], and amino sugar and nucleotide sugar metabolism [tre00520]. Statistical analysis showed that the differentially expressed genes were enriched for those involved in ribosome [tre03010] (FDR ≤ 0.05). It was interesting to note that the upregulated expression genes were enriched for sugar metabolism involved in citrate cycle (TCA cycle)[tre00020], glycolysis/gluconeogenesis [tre00010], pentose phosphate pathway [tre00030], carbon metabolism [tre01200], starch and sucrose metabolism [tre00500], and fructose and mannose metabolism [tre00051], although this enrichment was not statistically significant (FDR ≤ 0.05).

### qPCR

**Fig. 5:**
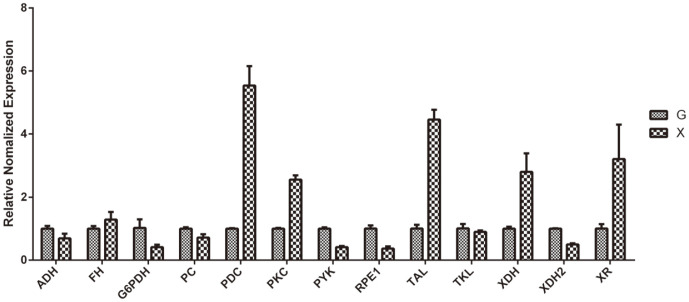
qPCR analysis of the selected genes. G: glucose. X: xylose. (XR: xylose reductase; XDH: xylitol dehydrogenase; G6PDH: glucose-6-phosphate dehydrogenase; TAL: transaldolase; TKL: transketolase; PYK: pyruvate kinase; PCK: phosphoenolpyruvate carboxykinase; PDC: pyruvate decarboxylase; RPE1: ribulose-5-phosphate 3-epimerase1; PC: pyruvate carboxylase; FH: fumarate hydratase; ADH: alcohol dehydrogenase)

In order to assess the accuracy of the RNA-Seq results, qPCR was implemented. Thirteen genes involved in different metabolism pathways were randomly selected (S1). The values shown are the mean values of three parallel experiments. As expected, the results of qPCR were consistent with those of RNA-Seq, indicating that the RNA-Seq results were credible (Fig. 5).

### Analysis of Genes Involved in Central Carbon Pathways

**Fig. 6:**
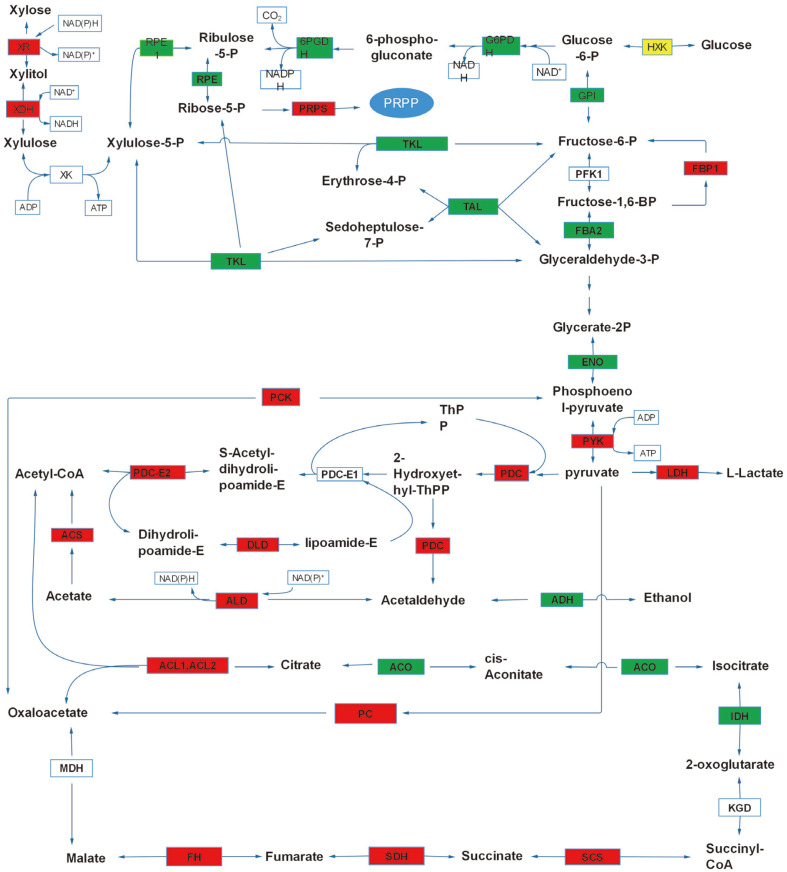
Expression profiles of genes involved in central carbon metabolism (including glycolysis, PPP, and TCA cycle) in HJ48 during xylose and glucose fermentation. The red boxes indicate transcriptional upregulation; the green boxes indicate transcriptional downregulation; the white boxes indicate no significant change in transcription; the yellow boxes indicate differences in the expression of isoenzymes.

The fermentation of xylose to ethanol is achieved in *T. reesei* via central carbon metabolism pathways that consist of the oxidoreductive pathway, the pentose phosphate pathway, glycolysis, and the ethanol fermentation pathway. To gain insight into the genes regulated by xylose, gene expression related to the central carbon metabolism was examined, using glucose as a control. As expected, there were a large number of differentially expressed genes between xylose and glucose (Table 4, Fig. 6).

Xylose must first be converted into xylulose, which is then phosphorylated by xylulokinase. Xylose reductase (XR), the first enzyme in the oxidoredutive pathway, is fundamental for xylose utilization [22]. Interestingly, the expression of xylose reductase and xylitol dehydrogenase (XDH) was found to be upregulated in this study. The xylulose kinase (XK) however, was not differentially expressed in *T. reesei* during xylose fermentation.

The pentose phosphate pathway is subdivided into two biochemical branches known as the oxidative and non-oxidative pentose phosphate pathway [23]. In the oxidative component of the pentose phosphate pathway, the enzymatic reactions are considered unidirectional. In order to help replenish NADPH-reducing equivalents, the enzymes glucose-6-phosphate dehydrogenase (G6PDH) and NADPH-producing 6-phosphogluconate dehydrogenase (6PGDH) are significantly upregulated with xylose in *S. cerevisiae* during the stress response[24]. However, we observed that the enzymes of the oxidative component of the pentose phosphate pathway were expressed at a relatively high level during glucose fermentation. The genes encoding the most important enzymes in the non-oxidative pentose phosphate pathway, the transaldolase (TAL) and transketolase (TKL), were significantly downregulated with xylose. In contrast, other enzymes in the non-oxidative pentose phosphate pathway, such as ribose 5-phosphate isomerase A, ribokinase, and ribose-phosphate pyrophosphokinase (PRPS), were expressed significantly higher with xylose compared to that with glucose.

The expression levels of several genes in the tricarboxylic acid cycles were upregulated with xylose compared to glucose. ATP-citrate synthase subunit 1 (ACL1) and ATP-citrate synthase subunit 2 (ACL2), which catalyze the condensation of acetyl coenzyme A and oxaloacetate to form citrate, were upregulated on xylose. Aconitate hydratase (ACO), which is repressed by ethanol, was expressed at a relatively low level during the xylose fermentation. The expression level of isocitrate dehydrogenase remained unchanged, whereas the expression of isocitrate dehydrogenase subunit 1 (IDH1), a mitochondrial NADP^+^-dependent enzyme, decreased with xylose. As for the KGD genes that encode 2-oxoglutarate dehydrogenase, the expression level of KGD1 and KGD2 encoding 2-oxoglutarate dehydrogenase E1 and 2-oxoglutarate dehydrogenase E2 components, respectively, were unchanged. Succinyl-CoA synthetase, various subunits of succinate dehydrogenase, as well as fumarate hydratase were all significantly upregulated with xylose. The expression level of cytoplasmic malate dehydrogenase (MDH), which is involved in the gluconeogenesis and glyoxylate cycles, was found to be unchanged. We also observed that genes encoding enzymes of the tricarboxylic acid cycle and respiration were upregulated during xylose metabolism. Moreover, Hap2, a subunit of the transcriptional activator complex Hap2/3/5, was upregulated with xylose under anaerobic conditions. In contrast, Hap3 was found to be upregulated during glucose fermentation.

We next analyzed the expression level of genes involved in glycolysis and alcohol fermentation during xylose and glucose fermentation. Kyoto Encyclopedia of Genes and Genomes (KEGG) analysis of hexokinases, which catalyze the conversion of glucose to glucose-6-phosphate, revealed that three genes were differentially expressed (TRIREDRAFT_73665, TRIREDRAFT_80231, TRIREDRAFT_79677). TRIREDRAFT_73665 and TRIREDRAFT_80231 were more highly expressed during xylose fermentation than during glucose fermentation. In contrast, the gene TRIREDRAFT_79677 was expressed more highly with glucose than with xylose. In *S. cerevisiae*, the expression of hexokinase1 and glucokinase was upregulated under aerobic and anaerobic conditions during xylose growth [25], and during growth on non-fermentable carbon sources [26]. The enzymes of glucose-6-phosphate isomerase (GPI) and fructose-1,6-bisphosphatase 1 (FBP1) are the major regulatory enzymes in the gluconeogenesis pathway. FPB1 was found to be upregulated in *T. reesei* during xylose fermentation, while GPI was downregulated with xylose. Pyruvate kinase (PYK), which is also known to be required in gluconeogenesis pathway, was found to be upregulated with xylose.

The expression levels of several enzymes in the lower half of the glycolytic pathway were unchanged, with the exception of enolase (ENO) and fructose-bisphosphate aldolase (FBA), which were downregulated, and pyruvate kinase and phosphoenolpyruvate carboxykinase (PCK), which were upregulated, with xylose compared to that with glucose. In the ethanol fermentation pathway, several genes showed significantly different expression. The isozymes of the pyruvate decarboxylase (PDC) gene TRIREDRAFT_121534 were upregulated with xylose, whereas expression of TRIREDRAFT_59267 was not significantly different. Transcripts of ADH, which encodes alcohol dehydrogenase, decreased with xylose. The result is consistent with our previous finding that only one alcohol dehydrogenase gene (TRIREDRAFT_22633), which catalyzes the reduction of acetaldehyde to ethanol, was upregulated in *T. reesei* during ethanol fermentation. Nevertheless, ethanol production is catalyzed by multiple alcohol dehydrogenase isozymes in *S. cerevisiae* [27]. Most of the genes involved in acetyl-CoA formation were upregulated during xylose fermentation, including pyruvate dehydrogenase E2 component (PDC-E2), dihydrolipoamide dehydrogenase (DLD), aldehyde dehydrogenase (ALD), and acetyl-CoA synthetase (ACS). In the pyruvate metabolism pathway, the expression of genes NAD-dependent malate dehydrogenase (MDH), fumarate hydratase (FH), and malate synthease (MS) was upregulated during xylose fermentation. Importantly, NAD-dependent malate dehydrogenase is the key enzyme involved in malate synthesis[28]. The increased expression of genes involved in malate synthesis suggests that the malate concentration is perhaps elevated during xylose fermentation.

### Analysis of Genes Involved in Sugar Transport

In the present study, several of the transporter genes in *T. reesei* were differentially expressed between xylose fermentation and glucose fermentation (Table 5). Among all the differentially expressed sugar transporter genes, 24 genes were more highly expressed during glucose fermentation, whereas 15 genes were more highly expressed during xylose fermentation. SNF3, a glucose receptor that generates a signal for induction of intracellular HXT expression, was downregulated with xylose. The glucose transporter Hgt-1 was upregulated with xylose. Rgt1, a transcription factor that regulates expression of HXT genes during glucose fermentation, was upregulated with glucose in *S. cerevisiae*.

## Discussion

We demonstrated from the genomic level the enormous advantages of using the filamentous fungus, *T. reesei*, over the popular yeast, *S. cerevisiae*, in ethanol fermentation from the pentose lignocellulosic sugar, xylose. Our *T. reesei* strain was able to utilize and ferment about 25 g/l of xylose, producing 1.1 g/l of ethanol under anaerobic condition after 72 h (Fig. 1). We attributed this successful xylose utilization/fermentation to the presence and expression of the essential genes responsible for adequate xylose utilization as sole carbon source in *T. reesei* (Figs. 5 and 6). Moreso, our *T. reesei* strain also wonderfully utilized and fermented glucose as carbon source, producing a peak ethanol concentration of 4.8 g/l from an initial 50 g/l glucose by 96 h (Fig. 1). These results therefore indicate the potentials and efficiency of *T. reesei* to both use hexose and pentose sugars in bioethanol production.

Xylose is converted to xylulose in filamentous fungi via the oxidoreductive pathway, which includes two reactions. First, xylose is reduced to xylitol by a NAD(P)H-dependent xylose reductase. Then, xylitol is oxidized to xylulose by a NADP^+^-dependent xylitol dehydrogenase [29, 30]. *S. cerevisiae* is unable to use xylose as a sole carbon source due to its lack of metabolic pathways for xylose utilization. This has led to the generation of several genetically modified strains of *S. cerevisiae* in recent years to increase the efficiency of xylose utilization, including expression of native pentose phosphate pathway enzymes such as xylose reductase and xylitol dehydrogenase [31, 32]. Unfortunately, the expression of these enzymes in these modified yeast strains has been generally poor. Yang *et al*. reported a promising transformation of the industrial *S. cerevisiae* KF-7 strain to utilizing xylose. Better xylitol production from xylose was recorded when the xylose reductase gene, *XYL1*, was overexpressed with lower xylose specificity. However, increasing the copy number of *XXL1* provided little improvement in xylitol production by the yeast and this was partially attributed to inadequate cofactor regeneration [33].

On the contrary, the genome of *T. reesei* contains all the genes for the metabolic pathways needed for xylose utilization. It is interesting to note that both xylose reductase and xylitol dehydrogenase were highly expressed in *T. reesei* during growth on xylose as sole carbon source in this study (Figs. 5 and 6). Similarly, the *T. reesei* strain created by Hong *et al*. with an increased copy number of xylose reductase gene was demonstrated to show higher reductase expression, unlike that of S. crevisiae, which consequently resulted in increased xylitol production and the amount of xylose consumed [34]. These results therefore highlight the enormous potentials of adopting *T. reesei* for consolidated bioprocessing.

The efficiency of the pentose phosphate pathway has been considered a barrier to efficient xylose utilization by *S. cerevisiae*, with the reactions of the non-oxidative pentose phosphate pathway catalyzed by the enzymes transaldolase and transketolase controlling the flux [35]. In addition, these enzymes regulate the switch between glycolysis and the pentose phosphate pathways [43]. The results presented here are consistent with previous observations, as the expression levels of transaldolase and transketolase were downregulated on xylose compared to those on glucose, which limited the rate of xylose metabolism [36]. Genetic methods to improve the rate of xylose utilization have focused primarily on enhancing the non-oxidative phases of the pentose phosphate pathway [37-39].

Kurylenko *et al*. reported that both transketolase and transaldolase were needed for xylose fermentation in *Ogataea polymorpha*, though both enzymes were not required for growth on xylose as a sole carbon source. Therefore, overexpression of transketolase and transaldolase elevated ethanol production from xylose in the *O. polymorpha* [40]. Overexpression of genes involved in non-oxidative pentose phosphate pathway, including *TAL1* and *TKL1*, also improved cell growth and increased the rate of xylose consumption in xylose-utilizing yeast strains [38]. In addition, it was also reported that the expression level of transketolase was upregulated through Msn2/4p-mediated stress responses, rather than in response to xylose as a sole carbon source in *S. cerevisiae* [11]. Overexpression of the XR/XDH pathway was utilized to engineer *S. cerevisiae* for xylose fermentation with xylose reductase using NAD(P)H as a cofactor and xylitol dehydrogenase, NAD^+^. In addition, these cofactors act greatly as limiting factors to xylose metabolism in transformed *S. cerevisiae*. However, we demonstrated that the expression levels of both xylose reductase and xylitol dehydrogenase were upregulated on xylose carbon source in *T. reesei* (Table 4, Fig. 6) thereby making this fungus an essential consolidated bioethanol producer from xylose. NAD(P)H is regenerated in *S. cerevisiae* mainly through the oxidative branch of the pentose phosphate pathway and the isocitrate dehydrogenases. Moreover, we found that the expression levels of glucose-6-phosphate dehydrogenase and 6-phosphogluconate dehydrogenase in the oxidative branch of the pentose phosphate pathway, and that of isocitrate dehydrogenase, were downregulated on xylose in *T. reesei*.

Jeppsson *et al*. had initially reported that ethanol yield in *S. cerevisiae* was increased by lowering the flux via the NADPH-producing pentose phosphate pathway [41]. This flux was lowered by disrupting both 6-phosphogluconate dehydrogenase and glucose-6-phosphate dehydrogenase. Decreasing the enzyme activity of phosphoglucose isomerase was also reported to lower the flux. Consequently, lowering the rate of the oxidative components of the pentose phosphate pathway led to reduced xylose uptake rate, attributed to the fact that limited NADPH-mediated xylose reduction induced the low rate of xylose fermentation [41].

Furthermore, Miskovic *et al*. also suggested that increasing the activity of phosphoglucose isomerase, which catalyzes the conversion of glucose-6-phosphate to fructose-6-phosphate, results in a reduced rate of xylose consumption [42]. This is because of the decreased reduction of glucose-6-phosphate to fructose-6-phosphate which consequently results in a reduced flux through the oxidative component of pentose phosphate pathway during glucose–xylose fermentation. This assertion is consistent with the experimental study by Jeppsson *et al*.[41], as lower phosphoglucose isomerase activity reduced the flux through the oxidative component of pentose phosphate pathway in batch fermentations with xylose as the sole carbon source [42]. Results from our study also concur with these two experimental studies regarding the effects of glucose-6-phosphate dehydrogenase, 6-phosphogluconate dehydrogenase, and phosphoglucose isomerase on xylose uptake rate in *T. reesei*. The expression levels of 6-phosphogluconate dehydrogenase, glucose-6-phosphate dehydrogenase, and glucose-6-phosphate isomerase were downregulated with xylose, resulting in a lower rate of xylose consumption [43] .

Many genes coding for enzymes of the tricarboxylic acid cycle were not repressed in glucose-rich medium. However, those genes were still expressed at a relatively high level in xylose-rich medium compared to glucose in our study (Figs. 5 and 6). Meanwhile, the genes encoding respiratory enzymes were also upregulated during xylose metabolism compared to glucose. The Hap complex consists of four subunits: *Hap2p, Hap3p, Hap5p* - each containing a highly conserved core domain required for DNA binding - and *Hap4p* (yeast) or HapX (filamentous fungi), which is a critical component of the transcriptional activator proteins [44-46]. In fungi, the Hap complex has been linked to fundamentally important biological processes, including nutrient acquisition, oxidative stress responses, and asexual reproduction [47]. In *S. cerevisiae*, the expression level of Hap4p was upregulated during xylose fermentation compared to that during glucose fermentation [11]. Nevertheless, the genes of *Hap2* and *Hap3* were expressed very differently in *T. reesei* between xylose and glucose fermentation, while *HapX* has not been found in *T. reesei* transcription (Fig. 6).

Zeng *et al*. showed that the expression of tricarboxylic acid cycle enzymes and respiratory enzymes was not repressed by xylose in transformed *S. cerevisiae* [29]. In that study, the expression level of *CIT2* and *CIT3*, which catalyze the condensation of acetyl coenzyme A and oxaloacetate, respectively, to form citrate, was upregulated during xylose fermentation, especially xylose as a sole carbon source. The transcripts of cytoplasmic malate dehydrogenase, which is involved in the gluconeogenesis and glyoxylate cycles, were also increased with xylose. These results are consistent with our findings and those of Matsushika *et al*. that growth of both yeast and filamentous fungi does not recognize xylose as a fermentable carbon source [11].

The transport of xylose into the cell is the initial rate-limiting step of xylose utilization [48, 49]. The predominant sugar transporters in *S. cerevisiae* are members of the HXT family [50]. These HXT genes belong to the major facilitator superfamily (MFS) of transporters [51]. Previous reports have demonstrated that MFS transporters display a higher affinity for glucose over xylose, thus contributing to limit pentose utilization during ethanol fermentation. Notably, xylose uptake in *S. cerevisiae* is very slow, and can be inhibited by glucose in the lignocellulosic sugar media. The use of engineered pentose transporters is therefore a promising approach to improve overall pentose uptake [52]. Nevertheless, very few pentose transporters have been functionally characterized and further research is needed to identify these targets. Filamentous fungi, on the other hand, possess a strong capacity to utilize lignocellulosic sugars. In fact, many pentose-assimilating fungal species possess specific transporters for pentose uptake. Several recombinant *S. cerevisiae* strains that were generated via introduction of heterologous xylose transporters have been reported to have significantly improved xylose uptake activity.

Jing *et al*. identified two novel xylose specific transporters, *An25* and *Xyp29*, from *Neurospora crassa* and *Pichia stipites*, respectively, and characterized them in *S. cerevisiae* at the molecular level [53]. Colabardini *et al*. discovered a high affinity xylose transporter *XtrD* in *Aspergillus nidulans*, which encodes an MFS transporter[49]. *S. cerevisiae* cells engineered to produce *XtrD* were capable of growth using xylose, glucose, galactose, and mannose as sole carbon sources, indicating that *XtrD* could transport multiple sugars. It was observed that expression of the *XtrD* transporter improved xylose uptake in a mutant *S. cerevisiae* strain.

Furthermore, transporter genes can be engineered to improve xylose uptake activity by introducing directed evolution. Using directed evolution, four novel xylose transporters that remained inhibited by glucose were created. Young *et al*. aimed to engineer hexose transporters with improved xylose uptake capacity [48]. Based on our transcriptome data, a number of sugar transporters were induced by xylose, indicating that the *T. reesei* genome may also possess high affinity xylose transporters. As of now, three *A. niger* (*XltA*, *XltB*, and *XltC*) and three *T. reesei* (*Str1*, *Str2*, and *Str3*) xylose transporters have been reported by Sloothaak *et al*. [54]. This result demonstrated that six transporters were able to transport xylose into yeast cells, but the substrate affinity and biochemical properties of these six transporters were significantly different with respect to their uptake of xylose. Amino acid sequence analysis showed the specific residues and motifs of transporters associated with xylose transporters. Specifically, *XltA* and *Str1* were induced by xylose and were dependent on the *XlnR/Xyr1* regulators, indicating that both of these transporters play a key role in xylose utilization.

Industrial microorganisms that can produce alcohol utilizing both hexose and pentose sugars simultaneously are essential for reducing the cost of lignocellulose conversion to bioethanol. *T. reesei* is considered as a candidate microorganism to be used industrially, although several limitations of its use exist currently. The present study provides the first transcriptomic comparison analysis of *T. reesei* exposed to either xylose or glucose. We revealed significant transcriptomic changes in genes involved in xylose metabolism and their significance. However, upstream regulation of these targets would demand further studies.

## Supplemental Materials



Supplementary data for this paper are available on-line only at http://jmb.or.kr.

## Figures and Tables

**Table 1:** Quantitative PCR primers of selected genes.

Gene name	Primer sequence
ADH-F	TGAGGAGTTCATCACGCACC
ADH-R	TGGCTCATCAGTAAGACCCG
FH-F	GTTTGAGGAGCACCTGGTCA
FH-R	GTTCTTGGCAACCTTGCTGG
G6PDH-F	TCCCCGAGTTCAAACCGAAG
G6PDH-R	GGCATAGCCGACGATCTTGA
PC-F	TGAGGAGTTCATCACGCACC
PC-R	TGGCTCATCAGTAAGACCCG
PKC-F	CGACAGCTTCCAGAAGGAGG
PKC-R	AGTCCCAAACGCTTTCCTCT
PDC-F	GCCGTCAAGACCAAGGAGTC
PDC-R	ACCCTCATACTACCCGGTCA
PYK-F	GGCATGGGCAACACAAACAC
PYK-R	GCCCCCGAGTTCCGTCATAA
RPE1-F	CATCAAGCCTGACACATCCG
RPE1-R	AGGTCGTTCCTTGGCATCA
TAL-F	TTCCGCTTCGACTTCAACGA
TAL-R	CTCCACAACTTACTGCCCCC
TKL-F	AGCAGTTTGGTCTCAACCGA
TKL-R	ACCCTTCCATTTTGCTCCCA
XDH-F	GATTTCGCCATCGACAACGG
XDH-R	ACTGACTCGCCATTCACTCG
XDH2-F	CGCGGCAATGGCACTATTC
XDH2-R	GTGTCGAAATTGAGCAGGGC
XR-F	GAACCTGGACAACACCTCCT
XR-R	ACAGGTAGAGCTTGTTGGCG
TUBULIN-F	AACTGTGAATGCCTCCAGGG
TUBULIN-R	TGCATAGACAAGGTGGCGTT

XR: xylose reductase; XDH: xylitol dehydrogenase; G6PDH: glucose-6-phosphate dehydrogenase; TAL: transaldolase; TKL: transketolase; PYK: pyruvate kinase; PCK: phosphoenolpyruvate carboxykinase; PDC: pyruvate decarboxylase; RPE1: ribulose-5-phosphate 3-epimerase1; PC: pyruvate carboxylase; FH: fumarate hydratase; ADH: alcohol dehydrogenase

**Table 2:** Summary of *T. reesei* HJ48 RNA-Seq data.

Sample name^1^	Raw reads	Clean reads	Clean bases	Error rate (%)	Q20 (%)^2^	Q30 (%)^2^	GC content (%)
G1	24594810	23954746	2.99G	0.01	97.37	93.56	57.56
G2	27760708	26974410	3.37G	0.01	97.05	92.83	57.47
G3	26704560	25965452	3.25G	0.01	97.37	93.38	58.27
X1	28509468	27703920	3.46G	0.01	96.98	92.6	58.32
X2	30946080	29993196	3.75G	0.01	96.93	92.57	58.75
X3	30787690	29958496	3.74G	0.01	96.91	92.36	57.6

1, G: glucose, X: xylose;“-1”,“-2” and “-3” represent biological replicates.

2, Q20 and Q30 indicate that the rates of correct base recognition were 99% and 99.9%, respectively.

**Table 3:** GO term enrichment (Corrected *p* value < 0.05) of genes with upregulated expression.

GO accession	Description	Term_type	Corrected *p* value^a^
GO:0005737	Cytoplasm	Cellular_component	1.77E-07
GO:0006412	Translation	Biological_process	1.04E-06
GO:0044444	Cytoplasmic part	Cellular_component	2.45E-06
GO:0003735	Structural constituent of ribosome	Molecular_function	3.42E-06
GO:0005840	Ribosome	Cellular_component	3.42E-06
GO:0043228	Non-membrane-bounded organelle	Cellular_component	0.000135
GO:0043232	Intracellular non-membrane-bounded organelle	Cellular_component	0.000135
GO:0032991	Macromolecular complex	Cellular_component	0.000135
GO:0030529	Ribonucleoprotein complex	Cellular_component	0.000179
GO:0005198	Structural molecule activity	Molecular_function	0.000935
GO:0044424	Intracellular part	Cellular_component	0.005238
GO:0044267	Cellular protein metabolic process	Biological_process	0.015116
GO:0005622	Intracellular	Cellular_component	0.015116
GO:0004298	Threonine-type endopeptidase activity	Molecular_function	0.039377
GO:0070003	Threonine-type peptidase activity	Molecular_function	0.039377
GO:0005623	Cell	Cellular_component	0.045453
GO:0044464	Cell part	Cellular_component	0.045453

^a^A hypergeometric test was used for statistical analysis, and *p*-values have been corrected for multiple testing by the Benjamini and Hochberg adjustment method. A corrected *p* value of < 0.05 is considered statistically significant.

**Table 4:** Expression levels of genes with significantly changed levels and expression in xylose metabolism relative to glucose metabolism.

Gene name	Description	log 2-fold change	Corrected *p* value
Pentose phosphate pathway			
*XYL2*	D-xylulose reductase A	+1.6145	3.58E-06
*DHSO*	Sorbitol dehydrogenase	+2.1999	4.81E-05
*XYL1*	NAD(P)H-dependent D-xylose reductase	+1.5904	0.025785
*F16P*	Fructose-1,6-bisphosphatase	+1.1311	8.97E-05
*GNTK*	gluconokinase	+2.2529	0.004768
*GNTK*	gluconokinase	-2.5238	4.51E-08
*GNL*	Gluconolactonase	+3.0605	0.000287
*RPIA*	Ribose-5-phosphate isomerase	+1.5554	0.016976
*KPR2*	Ribose-phosphate pyrophosphokinase 2	+2.3227	6.89E-05
*KPR5*	Ribose-phosphate pyrophosphokinase 5	+0.95173	0.015232
*KPR1*	Ribose-phosphate pyrophosphokinase 1	+1.6311	1.01E-06
*RBSK*	ribokinase	+2.606	1.30E-08
*6PGD1*	6-phosphogluconate dehydrogenase 1	-1.8057	3.33E-11
*RPE*	Ribulose-phosphate 3-epimerase	-1.1671	0.000492
*G6PD*	Glucose-6-phosphate 1-dehydrogenase	-1.893	4.25E-12
*G6PI*	Glucose-6-phosphate isomerase	-1.6248	1.18E-09
*TKT*	Transketolase	-0.65097	0.011435
*TAL*	Transaldolase	-1.6075	1.36E-09
*ALF*	Fructose-bisphosphate aldolase	-0.73024	0.004201
Citrate cycle (TCA cycle)			
*SDHB*	Succinate dehydrogenase	+1.0193	0.00076
*SDHB*	Succinate dehydrogenase	+3.0182	4.79E-22
*FUMH*	Fumarate hydratase	+1.9891	1.94E-12
*ACL1*	ATP-citrate synthase subunit 1	+5.3534	1.77E-62
*SUCB*	succinyl-CoA ligase [GDP-forming] subunit beta	-1.8567	4.11E-12
*PYC*	Pyruvate carboxylase	+4.349	2.32E-25
*ACL2*	ATP-citrate synthase subunit 2	+4.7538	3.59E-46
*ODP2*	Dihydrolipoyllysine-residue acetyltransferase	+1.0428	0.007641
*ACON*	Aconitate hydratase	-0.82864	0.002098
*PCKA*	Phosphoenolpyruvate carboxykinase [ATP]	+2.3988	3.41E-13
*DLDH*	Dihydrolipoyl dehydrogenase	+1.1836	1.79E-05
*IDH1*	Isocitrate dehydrogenase [NAD] subunit 1	-1.6149	1.10E-08
Glycolysis			
*KPYK*	Pyruvate kinase	+1.5263	3.01E-05
*ENO*	Enolase	-1.7957	1.85E-11
*ALDH*	Aldehyde dehydrogenase	-4.9115	3.82E-17
*HXK*	Hexokinase	+5.8109	7.78E-60
*HXKG*	Glucokinase	+3.1622	3.10E-13
*YLX7*	aldehyde dehydrogenase-like protein	+3.8971	9.83E-06
*GALM*	Aldose 1-epimerase	+1.552	0.003404
*HXK1*	Hexokinase-1	-2.2981	1.46E-14
*PDC*	Pyruvate decarboxylase	+1.1739	0.000111
*ACSA*	Acetyl-coenzyme A synthetase	+3.1111	1.67E-12
*FADH*	S-(hydroxymethyl)glutathione dehydrogenase	-2.9955	1.36E-26
*YMY9*	Glucose-6-phosphate 1-epimerase	+4.1848	1.34E-22
			
Pyruvate metabolism			
*CYB2*	Cytochrome b2	+5.263	2.98E-27
*KPYK*	Pyruvate kinase	+1.5263	3.01E-05
*ALDH*	Aldehyde dehydrogenase	-4.9115	3.82E-17
*MAOX*	NADP-dependent malic enzyme	+1.4446	0.00285
*THIL*	Acetyl-CoA acetyltransferase	-1.5502	2.97E-08
*HOSM*	Homocitrate synthase	-1.2422	0.001585
*ACYP*	Acylphosphatase	+2.3851	0.021204
*LDHD*	D-lactate dehydrogenase	+1.3151	0.017206
*MAOM*	NAD-dependent malic enzyme	-1.9184	1.20E-12
*THIL*	Acetyl-CoA acetyltransferase	-1.8059	3.91E-10
*ACAC*	Acetyl-CoA carboxylase	+4.6912	4.88E-34
*ACSA*	Acetyl-coenzyme A synthetase	+3.1111	1.67E-12
*CYB2*	Cytochrome b2	-4.3735	1.68E-06
*MASY*	Malate synthase	+3.7659	9.10E-24

^a^Log 2-fold change of differential expression; “+” means upregulated genes, “-” means downregulated genes. bA hypergeometric test was used for statistical analysis, and *p*-values have been corrected for multiple testing by the Benjamini and Hochberg adjustment method. A corrected *p* value of <0.05 is considered statistically significant.

**Table 5:** Differentially expressed genes, encoding transporters, between glucose and xylose in *T. reesei* HJ48.

Gene name	Description	Log 2-fold change^a^	Corrected *p* value^b^
*SNF3*	High-affinity glucose transporter SNF3	+4.0136	0.0050108
*MCH5*	Riboflavin transporter MCH5	+3.2995	0.0051801
*STL1*	Sugar transporter STL1	+3.8262	0.009133
*YHS6*	Uncharacterized MFS-type transporter	+0.78311	0.019183
*ESBP6*	Uncharacterized transporter ESBP6	+1.2191	0.015217
*MOT10*	Monocarboxylate transporter 10	+4.0177	0.000146
*MCH5*	Riboflavin transporter MCH5	+2.1355	0.001084
*MCH1*	Probable transporter MCH1	+3.1072	9.35E-07
*YKT8*	Uncharacterized MFS-type transporter	+3.1593	1.96E-11
*STL1*	Sugar transporter STL1	+6.5792	0.001033
*YGL4*	YEAST Putative oligopeptide transporter	+1.3784	5.37E-06
*RCO3*	Probable glucose transporter rco-3	+7.0732	1.74E-38
*HGT1*	High-affinity glucose transporter	+5.5797	2.49E-67
*YKT8*	Uncharacterized MFS-type transporter	+1.188	4.87E-05
*YHMA*	Uncharacterized MFS-type transporter	+2.8165	8.04E-09
*MOT12*	Monocarboxylate transporter 12	+1.8077	8.42E-07

^a^Log 2-fold change of differential expression; “+” means upregulated genes, “-” means downregulated genes.^b^A hypergeometrictest was used for statistical analysis, and *p*-values have been corrected for multiple testing by the Benjamini and Hochberg adjustment method. A corrected *p* value of <0.05 is considered statistically significant.
