# Supplementary material for: Transcription Analysis of Recombinant Trichoderma reesei HJ-48 to Compare the Molecular Basis for Fermentation of Glucose and Xylose
Source: J Microbiol Biotechnol. 2020 Jul 17;30(10):1467–79. doi: 10.4014/jmb.2004.04007 (PMC9745658; doi:10.4014/jmb.2004.04007)
Supplement: Supplementary file 1 [file JMB-30-10-1467-supple.pdf]

J. Microbiol. Biotechnol.
https://doi.org/10.4014/jmb.2004.04007

Supplemental File:

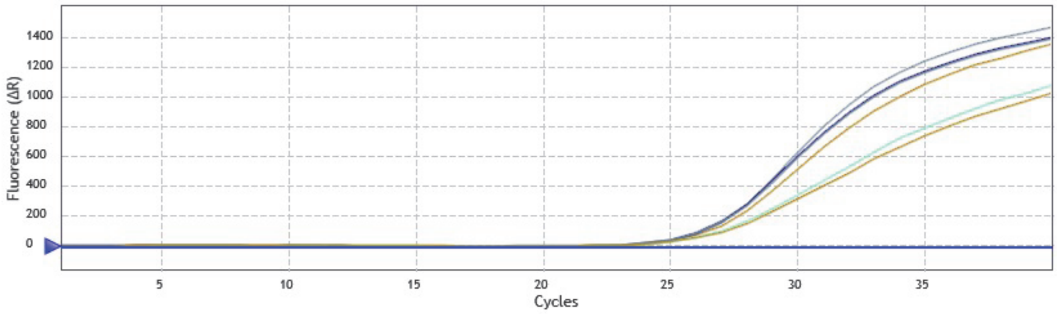

Fig1: the amplification curve of alcohol dehydrogenase (ADH)

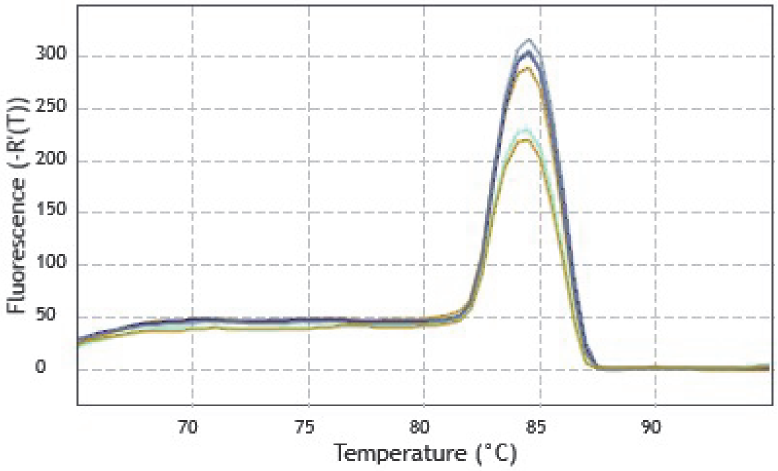

Fig2: the melt curve of alcohol dehydrogenase (ADH)

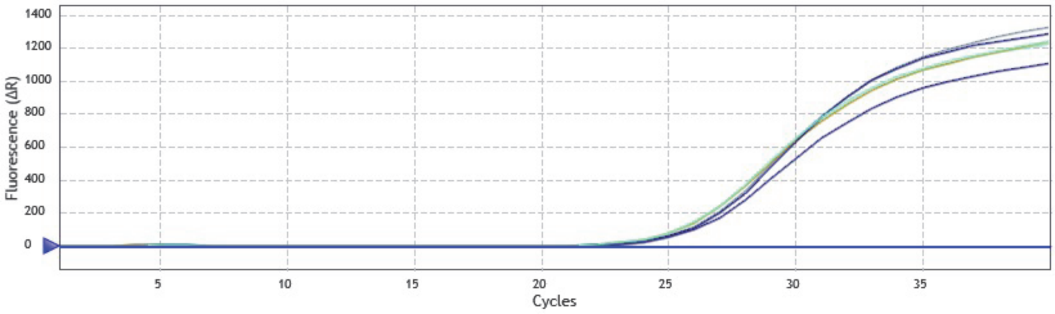

Fig3: the amplification curve of fumarate hydratase (FH)

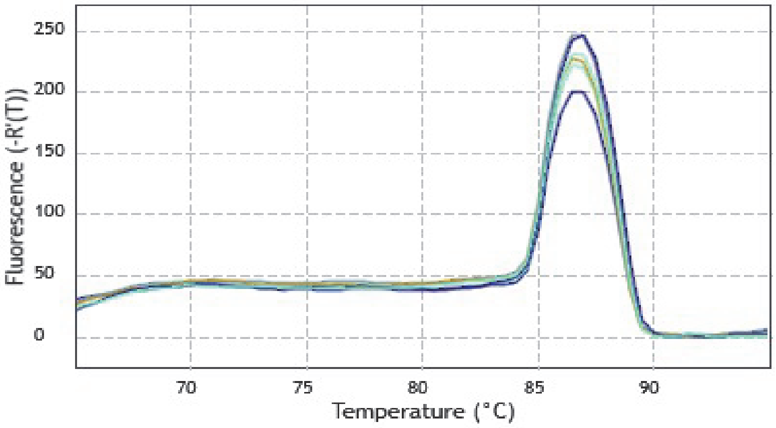

Fig4: the melt curve of fumarate hydratase (FH)

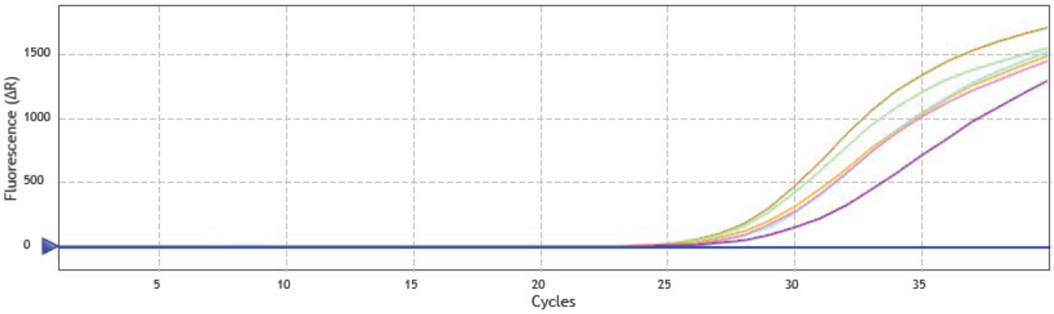

Fig5: the amplification curve of glucose-6-phosphate dehydrogenase（G6PDH）

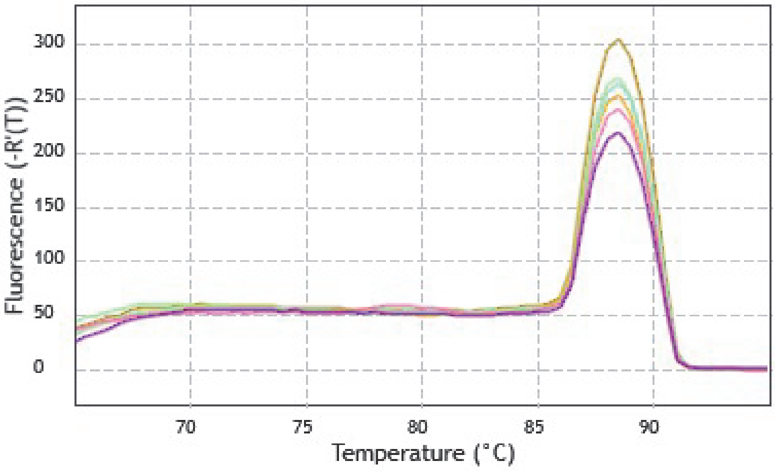

Fig6: the melt curve of glucose-6-phosphate dehydrogenase（G6PDH）

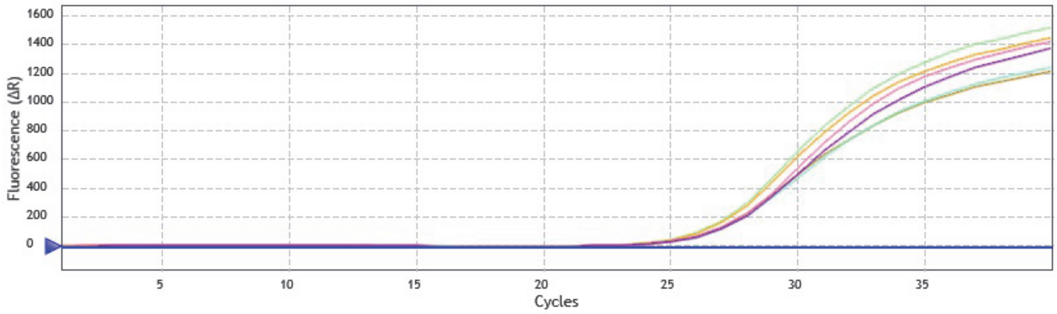

Fig7: the amplification curve of pyruvate carboxylase (PC)

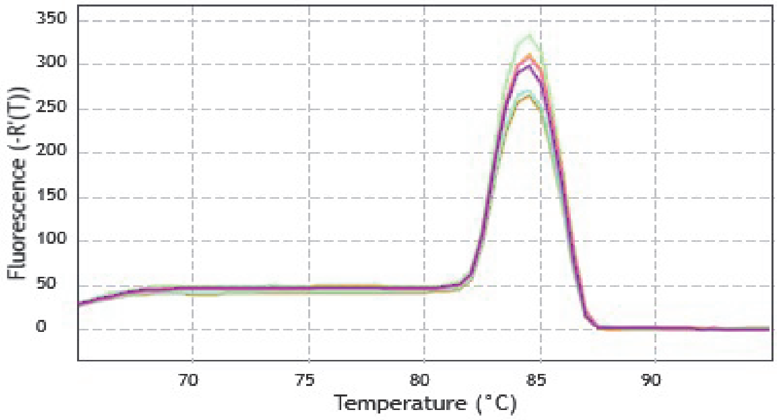

Fig8: the melt curve of pyruvate carboxylase (PC)

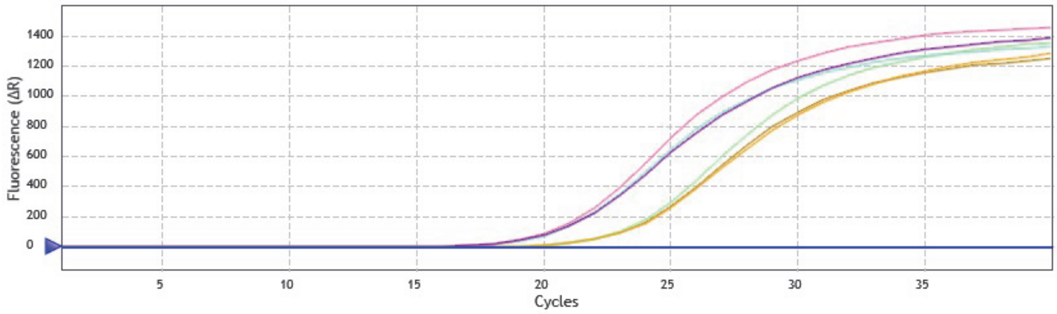

Fig9: the amplification curve of pyruvate decarboxylase (PDC)

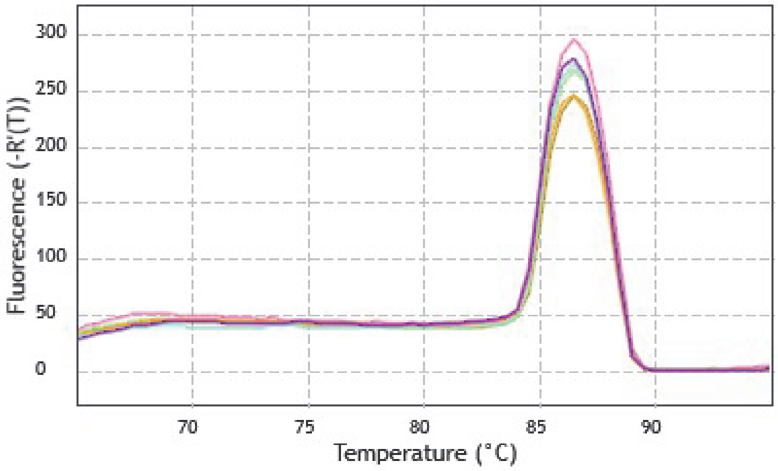

Fig10: the melt curve of pyruvate decarboxylase (PDC)

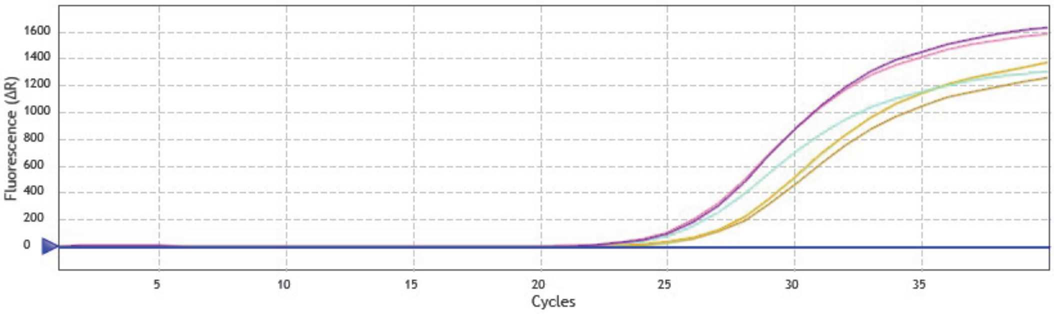

Fig11: the amplification curve of phosphoenolpyruvate carboxykinase (PCK)

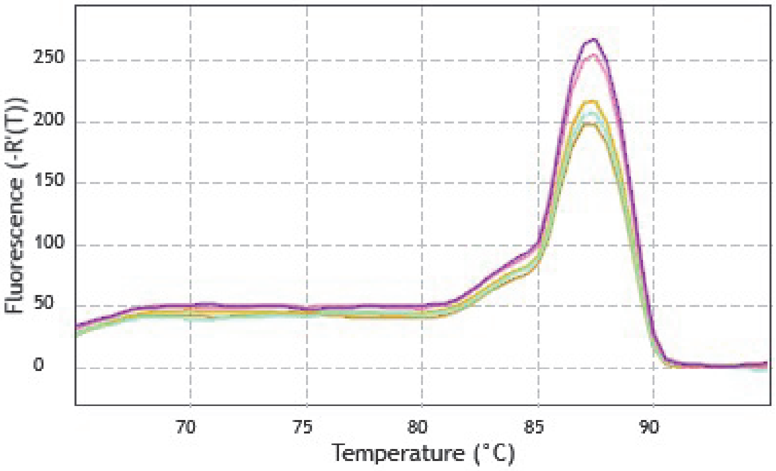

Fig12: the melt curve of phosphoenolpyruvate carboxykinase (PCK)

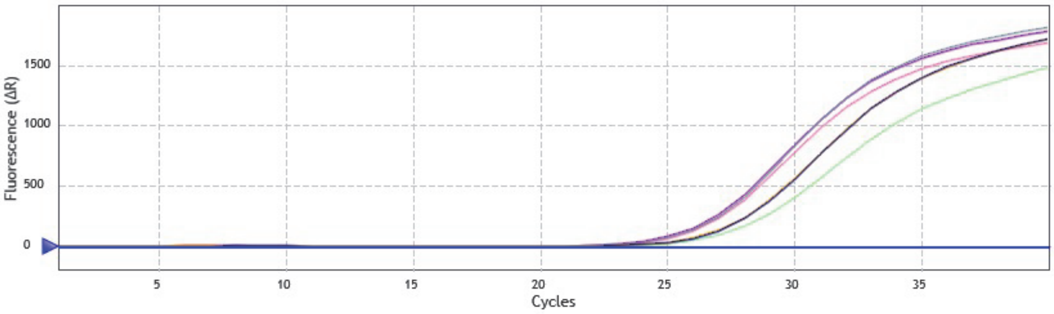

Fig13: the amplification curve of pyruvate kinase (PYK)

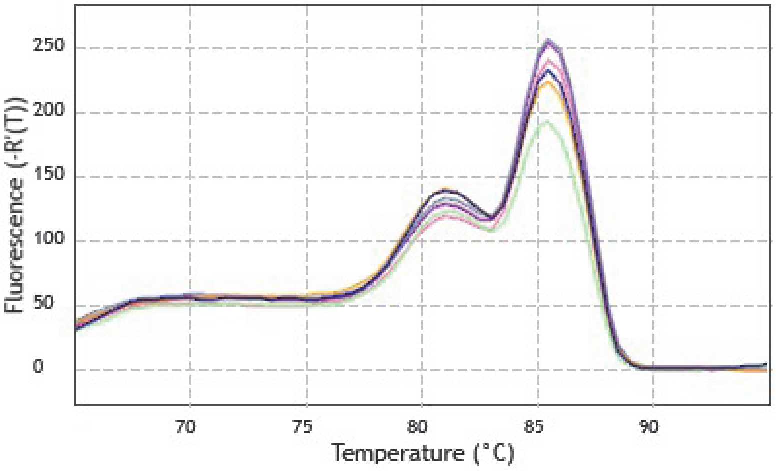

Fig14: the melt curve of pyruvate kinase (PYK)

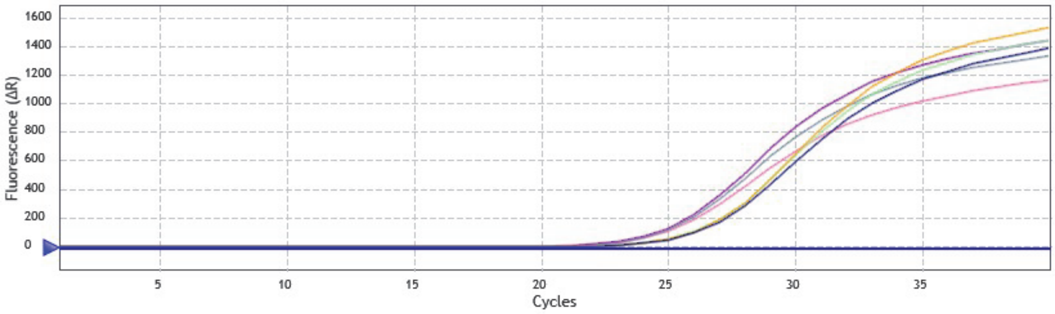

Fig15: the amplification curve of ribulose-5-phosphate 3-epimerase1 (RPE1)

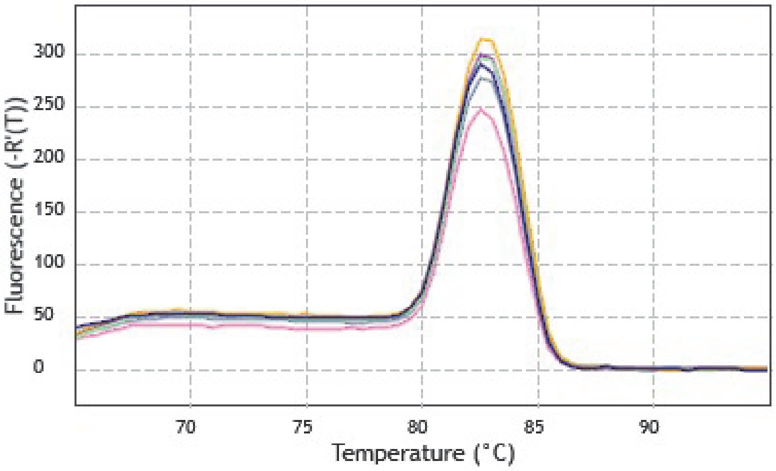

Fig16: the melt curve of ribulose-5-phosphate 3-epimerase1 (RPE1)

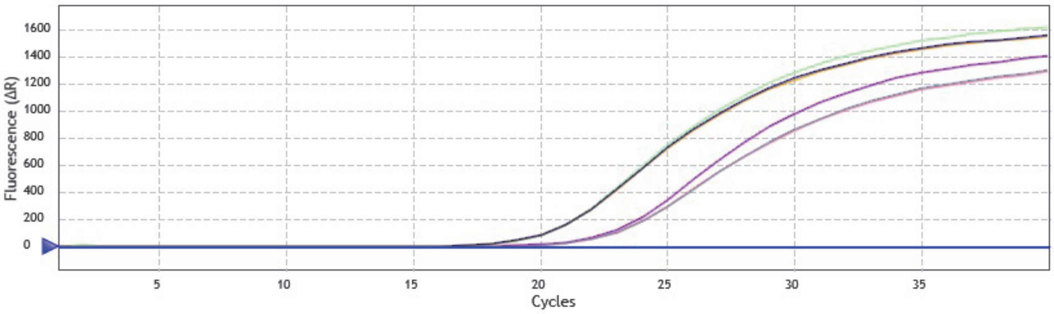

Fig17: the amplification curve of transaldolase (TAL)

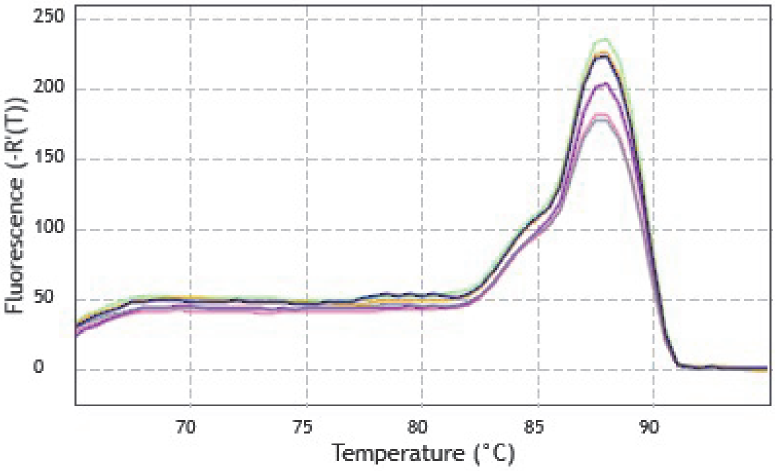

Fig18: the melt curve of transaldolase (TAL)

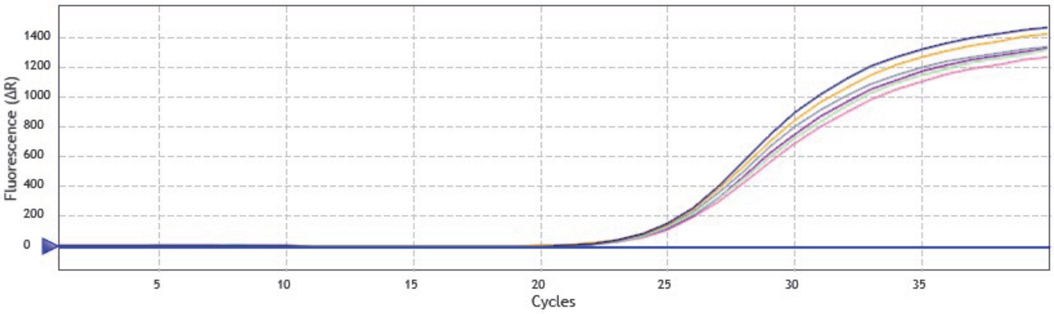

Fig19: the amplification curve of transketolase (TKL)

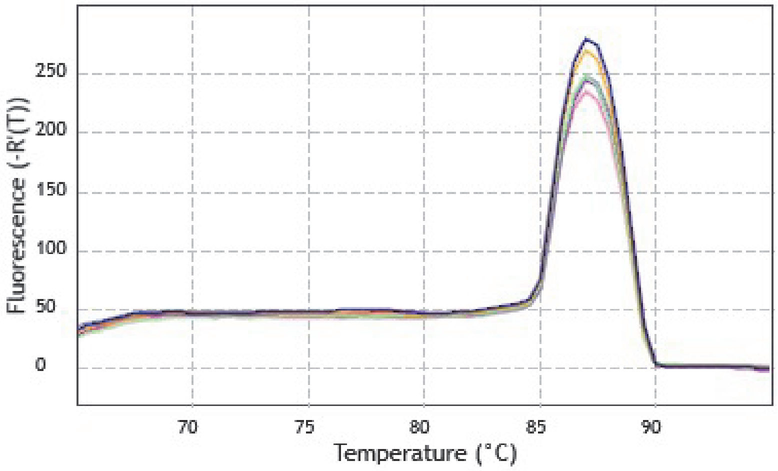

Fig20: the melt curve of transketolase (TKL)

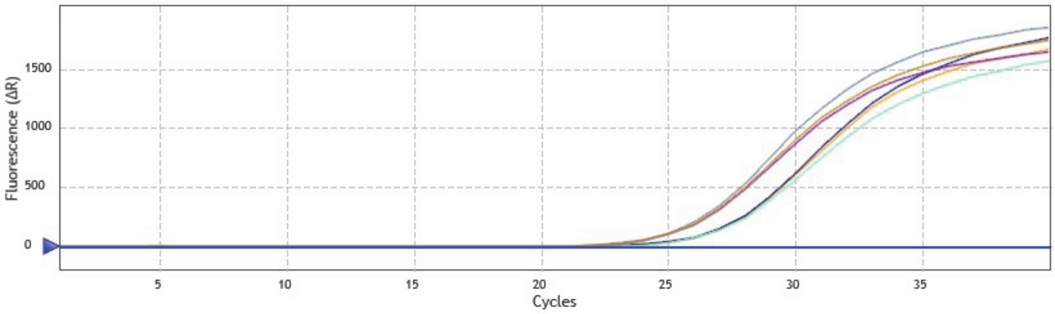

Fig21: the amplification curve of xylitol dehydrogenase (XDH)

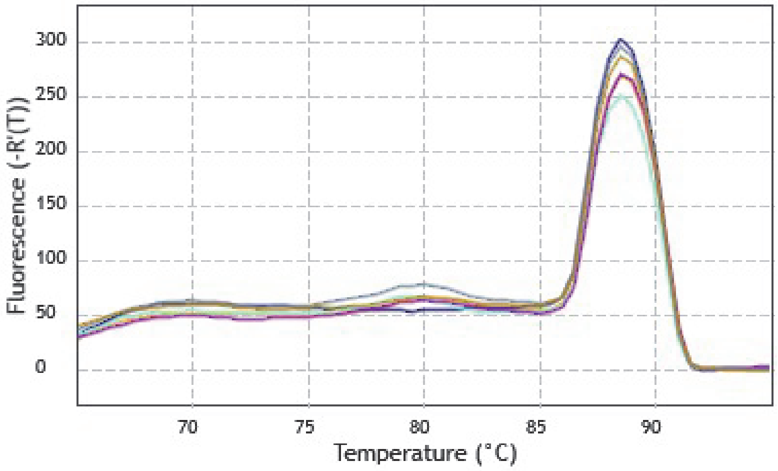

Fig22: the melt curve of xylitol dehydrogenase (XDH)

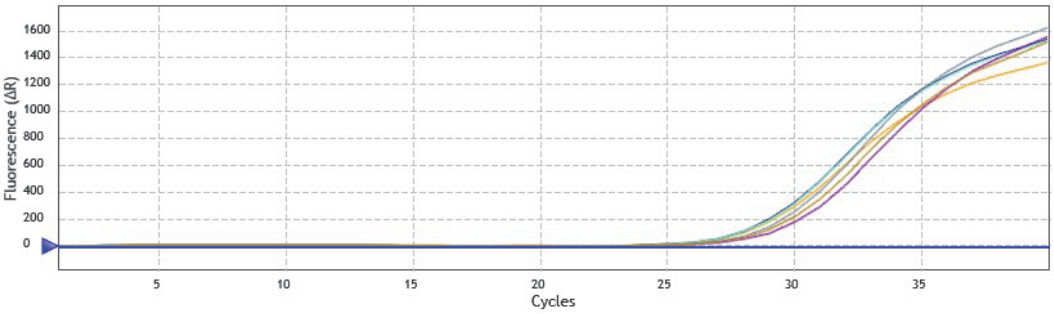

Fig23: the amplification curve of xylitol dehydrogenase 2 (XDH)

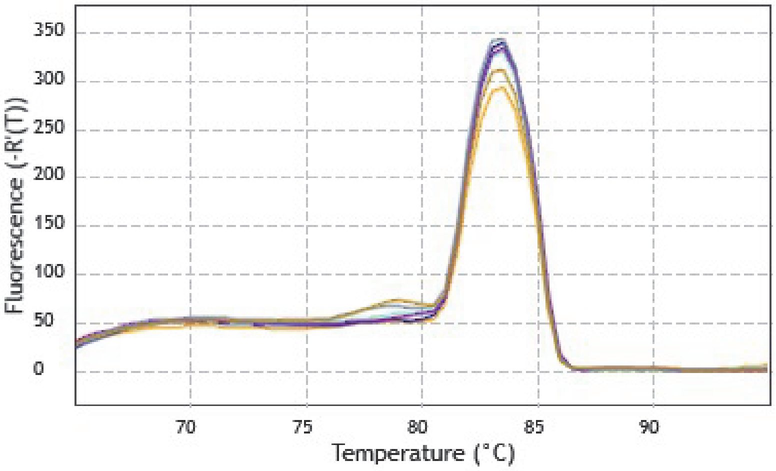

Fig24: the melt curve of xylitol dehydrogenase 2 (XDH)

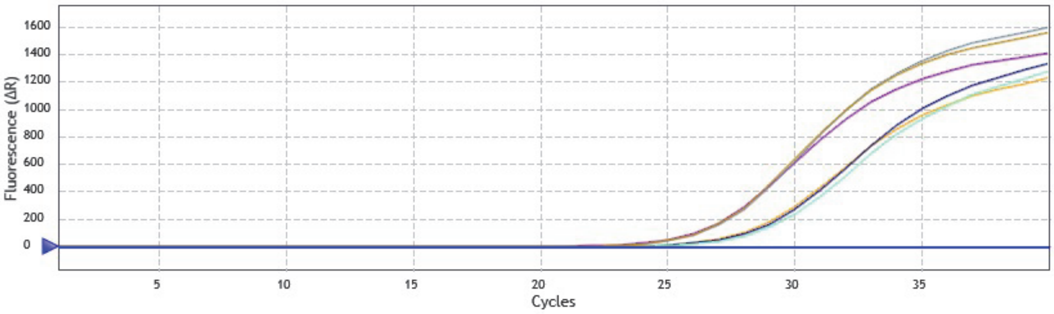

Fig25: the amplification curve of xylose reductase (XR)

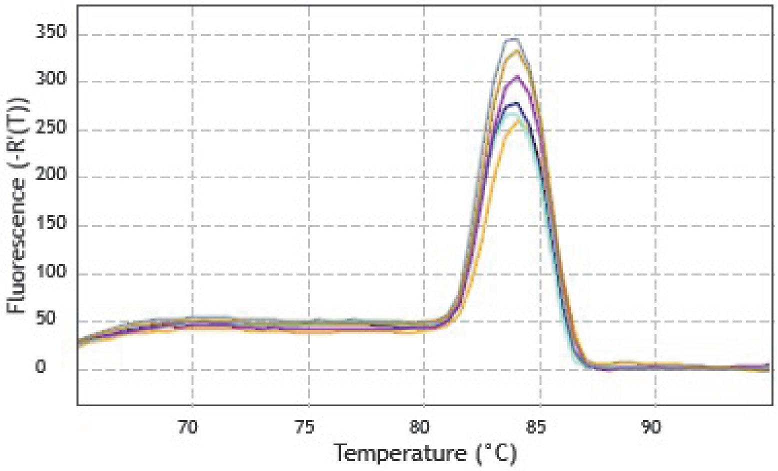

Fig26: the melt curve of xylose reductase (XR)

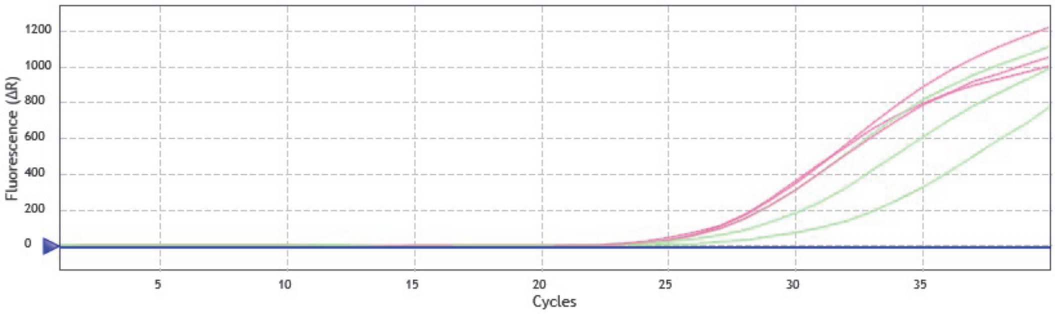

Fig27: the amplification curve of TUBULIN

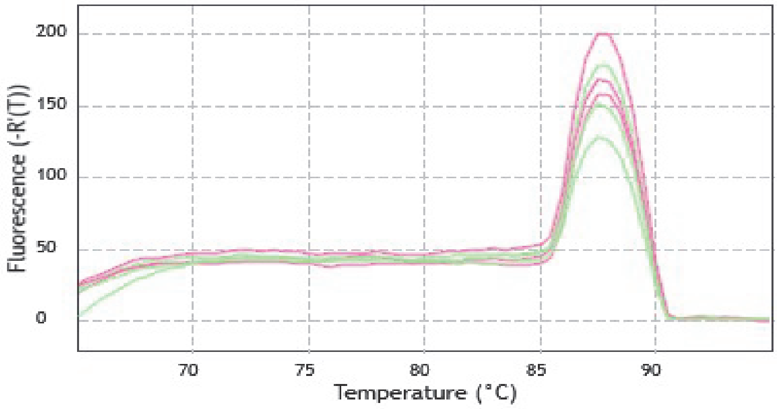

Fig28: the melt curve of TUBULIN
